# Supplementary material for: Decreased Fetal Movements: A Sign of Placental SARS-CoV-2 Infection with Perinatal Brain Injury
Source: Viruses. 2021 Dec 15;13(12):2517. doi: 10.3390/v13122517 (PMC8706116; doi:10.3390/v13122517)
Supplement: Supplementary file 1 [file viruses-13-02517-s001.zip › viruses-1502055-supplementary.pdf]

Supplementary Materials

This appendix has been provided by the authors to give readers additional information about their work.

Figure S1: CASE 1 - Fetal heart rhythm (FHR) monitoring at admission

Cardiotocogram showing reduced variability and no acceleration of the FHR.

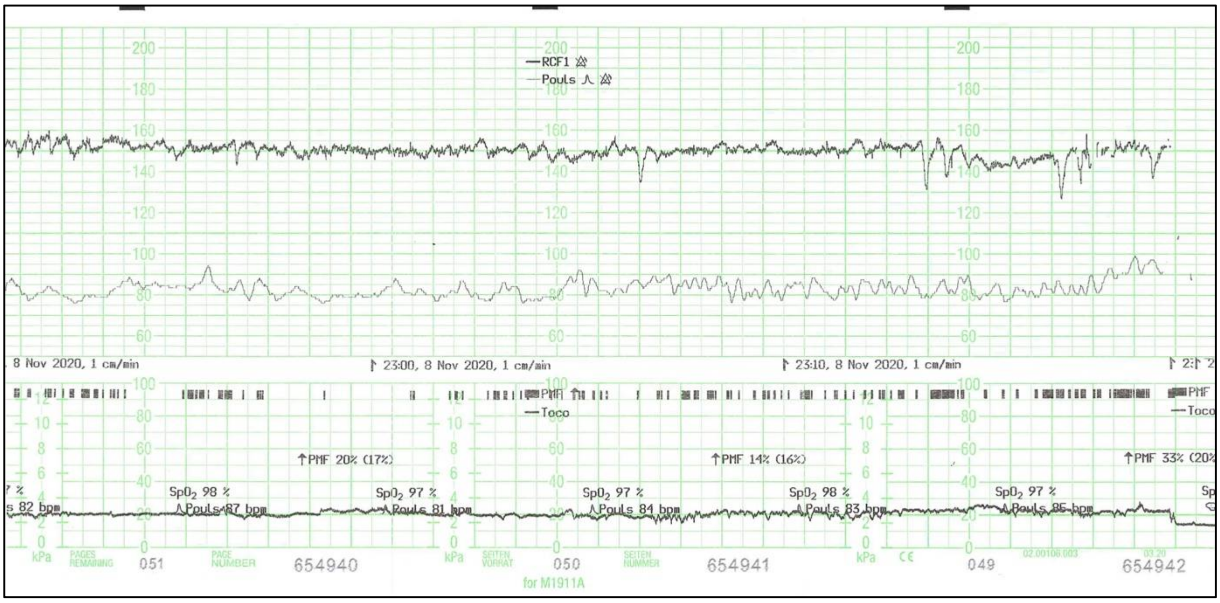

Figure S2: CASE 1 - Placental gross examination

Photograph of macroscopic sections of the placenta with frequent transplacental lattice-like depositions of fibrin, affecting more than 80% of the total placental volume.

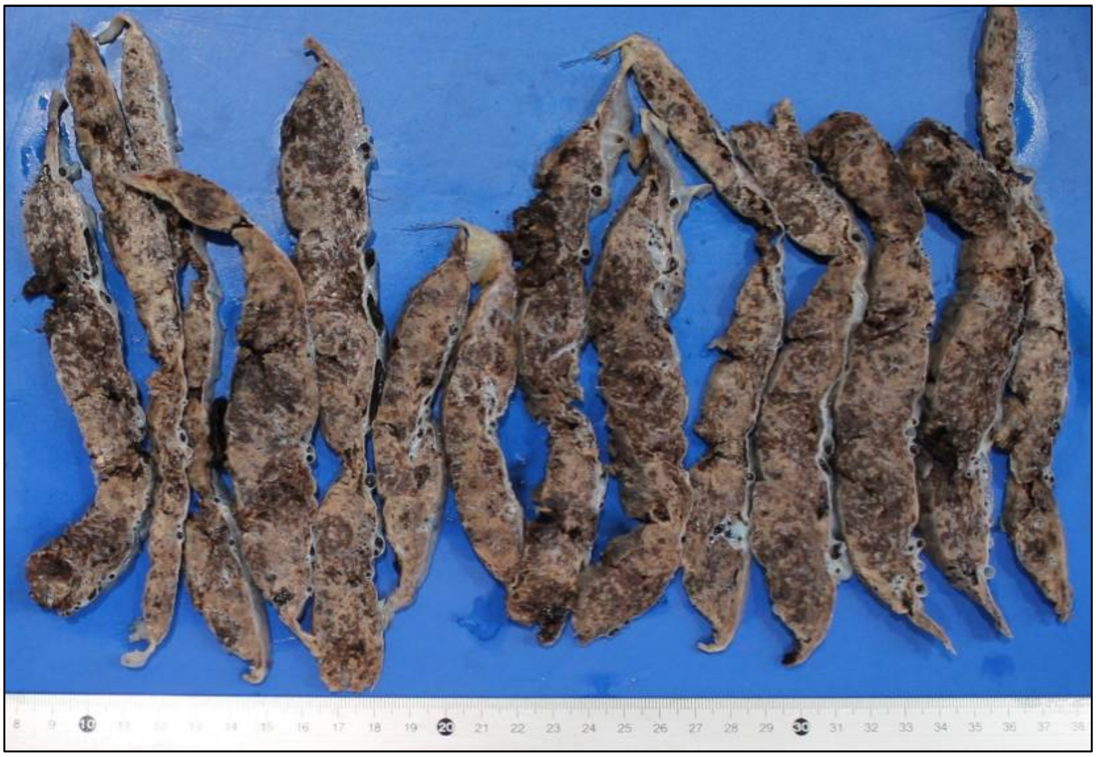

Table S1: Neonatal laboratory values at birth

**Blood chemistry**	**Case 1**	**Case 2***	Reference range and unite of
Sodium	142	136	135-145 mmol/l
Potassium	3.7	4.2	3.5-4.5 mmol/l
Magnesium	0.78	0.64	0.65- 1.10 mmol/l
Phosphate	0.94	1.03	1.60- 3.10 mmol/l
Urea	6.7	3.5	1.1-6.8 mmol/l
Creatinine	83	86	21-75 mcmol/l
C reactive protein	-	1	< 10 mg/l
Aspartate aminotransferase	-	4645	9-45 U/l
Alanine aminotransferase	-	659	48-406 U/l
γ- GT	-	153	6-42 U/l
Total serum bilirubin	54	51	mcmol/l

Conjugated bilirubin	4	2	mcmol/l
**Blood count**			
White cell count	15. 1	14.6	5.0-20.0 G/l
Red cell count	3.53	4.29	3.6- 6.2 T/l
Haemoglobin	137	162	125-205 g/l
Haematocrit	45	50	39-63 %
Platelet count	272	127	150-350 G/l
Erythroblasts	185	116	/100 leucocytes
Neutrophils	3.47	8.47	1.0-9.5 G/l
Lymphocytes	10.72	5.26	2.0-17 G/l
**Haemostatic parameters**			
Prothrombin time	-	10% (51.4	80-120%
INR	-	5.2	
Activated partial	-	110	26-37 sec
Thrombin time	-	33	14-19 sec
Fibrinogen	-	< 0.3	2.0-4.0 g/l
**Blood gas analyse**	Venous	Arterial	
	FiO2 0.4	FiO2 1	
pH	7.20	7.02	
PCO2	39.5	36	mmHg
PO2	42.6	71.8	mmHg
Bicarbonate	15.1	9.3	mmol/l
SatO2	83.9	96.4	%
Anionic gap	15.9	16.7	mmol/l
Lactate	9	16	mmol/l

* : patient was born in a peripheral hospital and laboratory findings are those at NICU admission of University Hospital of Lausanne

- : no values
